# Supplementary material for: Efferent feedback controls bilateral auditory spontaneous activity
Source: Nat Commun. 2021 Apr 27;12:2449. doi: 10.1038/s41467-021-22796-8 (PMC8079389; doi:10.1038/s41467-021-22796-8)
Supplement: Supplementary file 1 — Supplementary Information [file 41467_2021_22796_MOESM1_ESM.pdf]

Supplementary Information

Efferent feedback controls bilateral auditory spontaneous activity

Yixiang Wang[1], Maya Sanghvi[1], Alexandra Gribizis[1,5], Yueyi Zhang[1], Lei Song[2,7], Barbara Morley[6], Daniel G. Barson[1], Joseph Santos-Sacchi[1,2,3], Dhasakumar Navaratnam[1,2,4], Michael Crair[1,8,#]

1 Department of Neuroscience, Yale University School of Medicine, New Haven, CT 06510, USA

2 Department of Surgery (Otolaryngology), Yale University School of Medicine, New Haven, CT 06510, USA

3 Department of Cellular and Molecular Physiology, Yale University School of Medicine, New Haven, CT 06510, USA

4 Department of Neurology, Yale University School of Medicine, New Haven, CT 06510, USA

5 Max Planck Florida Institute for Neuroscience, One Max Planck Way, Jupiter, FL 33458, USA

6 Center for Sensory Neuroscience, Boys Town National Research Hospital, Omaha, NE 68131, USA

7 Department of Otolaryngology-Head and Neck Surgery, Shanghai Ninth People's Hospital, Shanghai Jiao Tong University School of Medicine, Shanghai 200011, China

8 Kavli Institute for Neuroscience, Yale University, New Haven, CT 06510, USA

#Correspondence: michael.crair@yale.edu

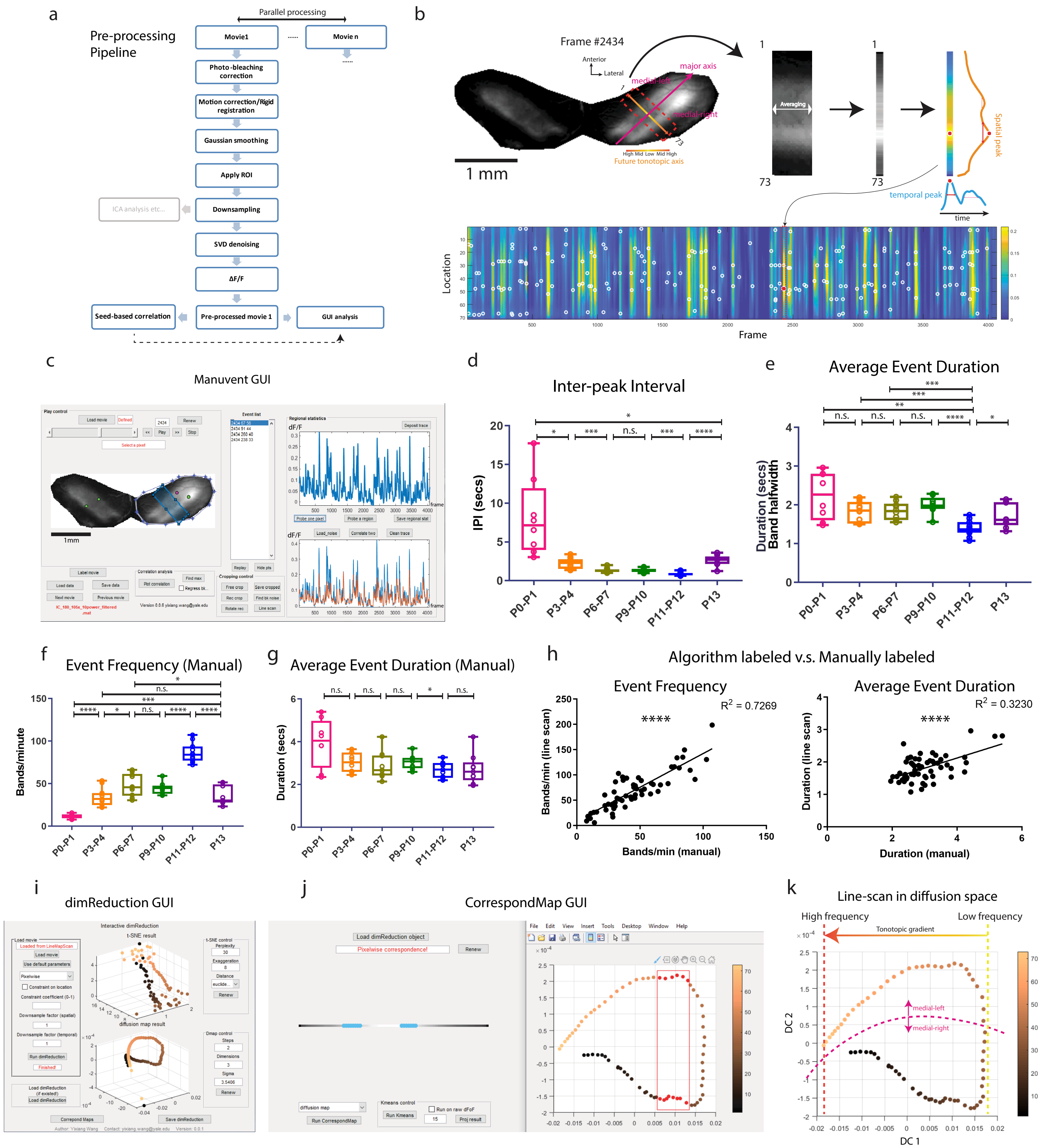

Supplementary Fig. 1: Software tools and further analysis of sptiotemporal properties of spontaneous activity (related to Fig. 1 and Methods)

Supplementary Fig.1. Software tools and further analysis of spatiotemporal properties of spontaneous activity (related to Fig. 1)

a. Object-oriented pre-processing pipeline for high throughput parallel processing
b. Major steps to generate "line-scans" across the future tonotopic axis. Top panels: Left-most image shows an example frame from a continuous movie with an ROI mask over the IC. Magenta arrow represents the major axis of spontaneous bands' intensity profile. Medial-left and Medial-right regions represent two future tonotopic domains on the opposite sides of the major axis. Yellow to orange gradient denotes the future tonotopic axis/reversal. Dashed red line indicates the rectangular ROI defined for averaging. Averaged one-dimensional line scan is shown in gray and parula color maps. Red dot denotes the intensity peak identified in the line-scan. Orange curve: spatial intensity distribution. Blue curve: temporal intensity trace. Double-headed red arrows on the curves represent half widths of spatial or temporal peaks. Bottom panel: a series of "line-scans" representing a continuous movie. The line scan on the top panel is enclosed in a dashed rectangle. The red dot denotes the same peak as shown above. All other peaks were labeled as empty with circles. Colormap: parula (MATLAB). Scale bar indicates 1 mm. 1 and 73 denotes the first and last pixels. See also Methods.
c. Snapshot of "Manuvent" GUI. Showing the same example movie as in Fig.1.
d. Average inter-peak interval in seconds across age groups. Defined as the mean temporal interval between neighboring peaks in the line-scan analysis.
e. Average event duration across age groups. Defined as the mean temporal half width of all fluorescent peaks in the line-scan analysis.
f. Event frequency across age groups (manually labeled). Measure by number of spontaneous bands/events observed per minute in both hemispheres of the IC by a human tester.
g. Average event duration across age groups (manually labeled). Duration is defined as the number of frames in between the first and last frames of events divided by acquisition frequency (10Hz).
h. Linear plots comparing algorithm-generated line-scan results (y axis) and manually labeled results (x axis). Data points include all movies used in Fig.1. **** $p < 0.0001$ (slope against zero: $F = 148.6$ (event frequency); $F = 26.72$ (duration))

i. Snapshot of "dimReduction" GUI. Showing the same dimensionality reduction result in Fig.1G but with three diffusion components. Also showing dimensionality reduction results based on t-SNE. See also Methods.

j. Snapshot of "CorrespondMap" GUI. Showing the same diffusion map result in Fig.1G. Selected points (red) in the right panel correspond to pixels on the line-scan map in the left panel (light blue). See also Methods.
k. Example diffusion map result. Pixels are projected to points in the diffusion space. DC 1/ DC 2: diffusion component 1/2. Single-headed yellow-to-orange arrow denotes the putative future tonotopic gradient. Dashed magenta curve delineates the boundary between the two symmetrical tonotopic domains: medial-left and medial-right. Colorbar denotes pixel number. Colormap: copper (MATLAB).

For all Box plots in this study: hinges: 25 percentile (top), 75 percentile (bottom). Box whiskers (bars): Max value (top), Min value (bottom). The line in the middle of the box is plotted at the median. Significance marks: n.s. $p > 0.05$, * $p < 0.05$, ** $p < 0.01$, *** $p < 0.001$, **** $p < 0.0001$, two-tailed unpaired t test with Welch's correction. Number of animals: P0-P1 (N = 8); P3-P4 (N = 11); P6-P7 (N = 10); P9-P10 (N = 9); P11-P12 (N = 11); P13 (N =9). Same animals as in Fig.1. Source data and exact *p* values are provided as a Source Data file.

Text for Supplementary Fig.1i-k:

We were also interested in whether the intrinsic dynamics of the spontaneous activity were able to reflect distinct functional organizations of the IC observed in mature animals. To address this question, we took advantage of dimensionality reduction and unsupervised learning techniques. High-dimension calcium imaging data was embedded to low-dimensional manifolds using diffusion map. Pixels on the line-scans differentiated as two mirroring domains along the vertical direction in the diffusion space (divided by a dashed magenta curve, Supplementary Fig.1k). When slicing the data points horizontally, we were able to extract symmetrically distributed pixels of similar future characteristic frequencies (Supplementary Fig.1j). This is consistent with the reverse organization of dual tonotopic maps in the DCIC and LCIC [23]. The distribution of functional clusters was mirror-symmetrical (Supplementary Fig.1k), similar to the diffusion map result.

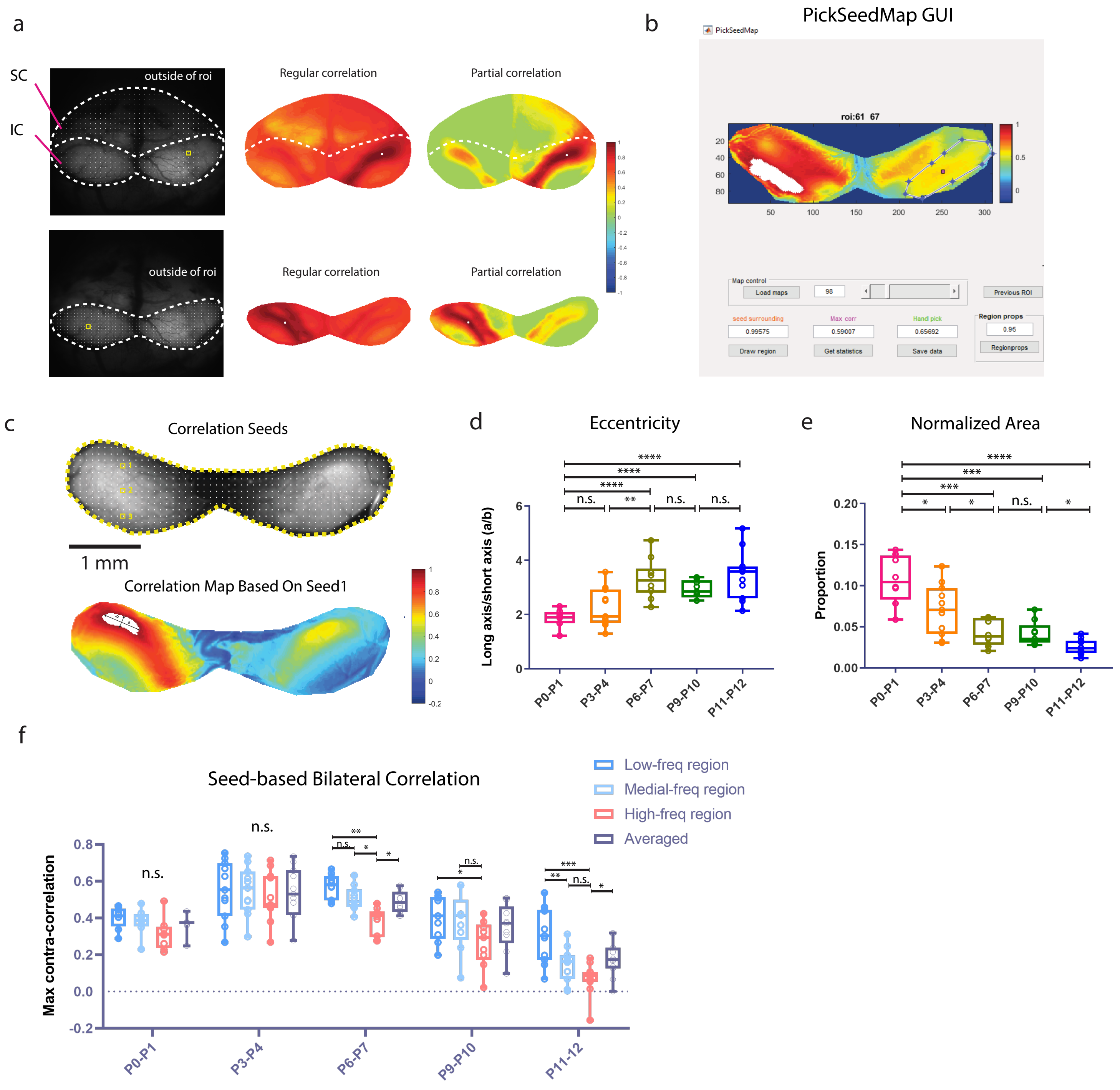

Supplementary Fig. 2: Demonstration of partial correlation (related to Fig. 1 and Methods)

Supplementary Fig.2. Demonstration of partial correlation (related to Fig.1)

a. Top left panel: Example correlation seeds covering the whole midbrain. Dashed white line delineates the boundary of the ROI mask. Each white dot inside the ROI denotes a reference seed. SC: superior colliculi. IC: inferior colliculi. The seed used for the example correlation maps is labeled with an empty yellow square; Top middle panel: correlation map generated with normal Pearson correlations; Top right panel: correlation map generated with partial correlations controlling on mean activity from pixels outside the ROI. Bottom panels: similar as the top panels but with a ROI mask over the IC only. Note that the color limit here is different from the color limit of all correlation maps in main figures. Colormap: jet (MATLAB). Colorscale: [-1, 1]. See also Methods.
b. Snapshot of "PickSeedMap" GUI. Used for correlation seeds selection and max correlation quantification in the contralateral hemisphere.
c. Top panel: Example of correlation seeds covering the whole IC. Each white dot denotes a reference seed. Three representative seeds used to generate representative correlation maps in Fig.1J are labeled with empty yellow squares and numbers 1,2,3. Bottom panel: a correlation map based on seed number 1. The white region denotes the binary connected component used for ellipse fitting with major (a) and minor (b) axes shown as black double-headed arrows. Colormap: jet (MATLAB). Colorbar: [-0.2, 1].
d. Eccentricity of fitted ellipses. Defined as the ratio of long axis (a) and short axis (b)
e. Area of fitted ellipses normalized by the size of hemispheres.
f. Fig.1k regrouped by ages to illustrate differences among regions within same age groups.

Number of animals: P0-P1 (N = 8); P3-P4 (N = 11); P6-P7 (N = 10); P9-P10 (N = 9); P11-P12 (N = 11). Same animals as in Fig.1. Significance marks: n.s. $p > 0.05$, $* p < 0.05$, $** p < 0.01$, $*** p < 0.001$, $**** p < 0.0001$, two-tailed unpaired t test with Welch's correction. Source data and exact p values are provided as a Source Data file.

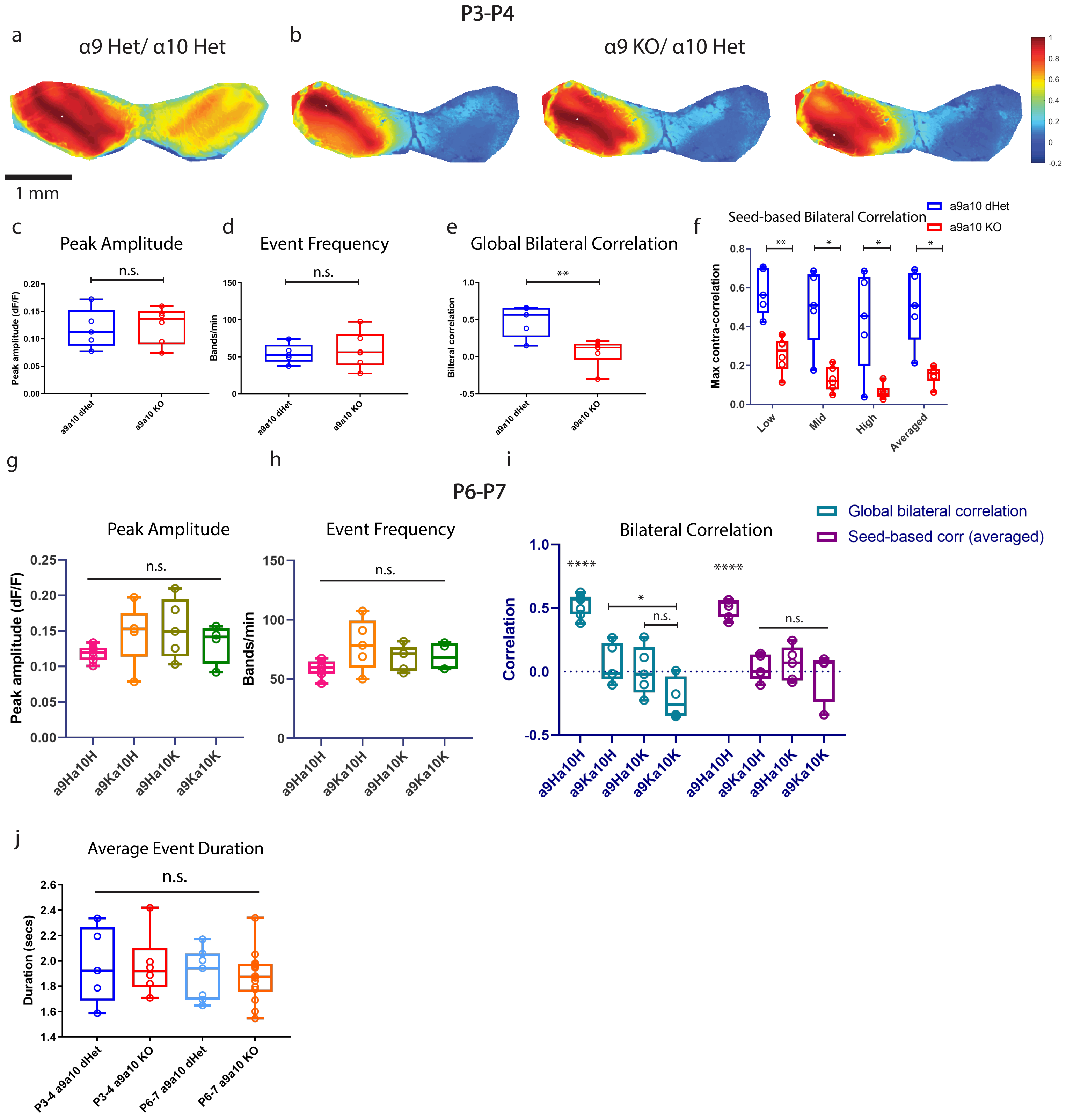

Supplementary Fig. 3: Further analysis on α9/α10 nAChR knouckouts (related to Fig. 2)

Supplementary Fig.3. α9/α10 nAChR knockouts lack bilateral coupling of spontaneous activity at P3-P4 (Related to Fig.2)

a. Example maps showing correlation patterns in an α9/α10 double heterozygous at P3.
b. Example maps showing correlation patterns in a α9 KO α10 Het littermate at P4.
c. Average peak amplitude. Similar to Fig.1d.
d. Event frequency. Similar to Fig.1e.
e. Global bilateral correlation. Similar to Fig.1j.
f. Seed-based bilateral correlation. Similar to Fig.1k.
g. Average peak amplitude (P6-7). Similar to Fig.1d.
h. Event frequency (P6-7). Similar to Fig.1e.
i. Global and seed-based bilateral correlation of different genotype groups. "Averaged" seed-based correlation is defined as the mean correlation averaged over the three regions.
j. Average event frequency across ages and genotypes. Similar to Supplementary Fig. 1e.

H: heterozygous; K: knockout. For instance, α9H α10K indicates the α9 heterozygous α9 10 knockout. Number of animals (P3-4): α9/α10 dHet (N = 5); α9/α10 KO (N = 6). Number of animals (P6-7): α9Het α9Het (N = 7); α9KO α10Het (N = 5); α9Het α10KO (N =5); α9KO α10KO (N = 4). All at P6-P7. Significance marks: n.s. $p > 0.05$, ∗ $p < 0.05$, ∗∗ $p < 0.01$, ∗∗∗ $p < 0.001$, **** $p < 0.0001$, two-tailed unpaired t test with Welch's correction. Source data and exact p values are provided as a Source Data file.

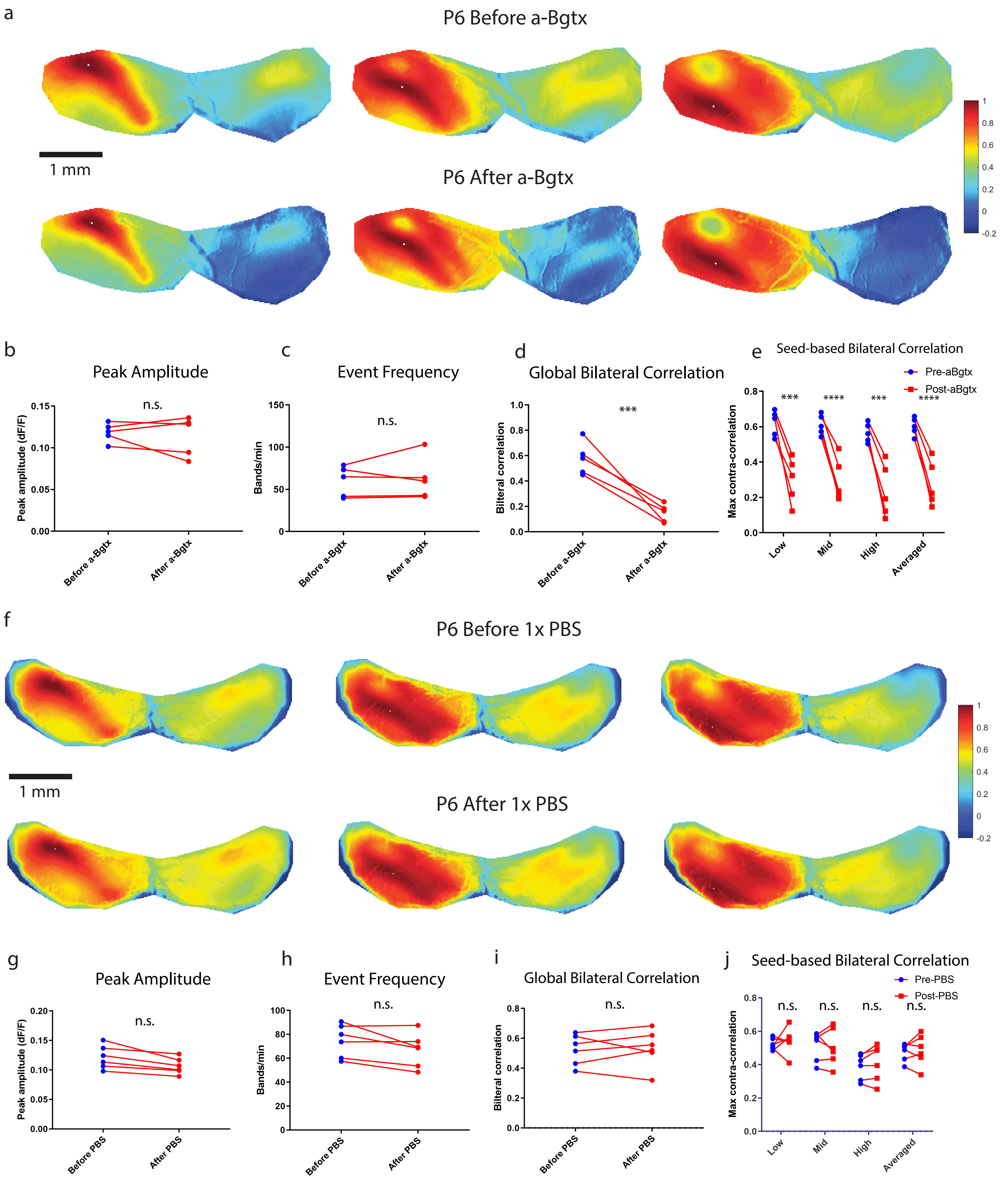

Supplementary Fig. 4: Acute alpha-bungratoxin application abolishes bilateral coupling in vivo (related to Fig. 3)

Supplementary Fig.4. Acute alpha-bungarotoxin application abolishes bilateral coupling in vivo (Related to Fig.3)

a. Example correlation maps showing correlation patterns in the IC before and after alpha-bungarotoxin from the same SNAP25-G6s animal at P6. Top panels: before apamin. Bottom panels: after apamin. Three typical representative seeds (white dots) located in the future low-, mid-, and high-frequency regions. Similar to Fig.3e.
b. Average peak amplitude. Similar to Fig.1d.
c. Event frequency. Similar to Fig.1e.
d. Global bilateral correlation. Similar to Fig.1j.
e. Seed-based bilateral correlation. Similar to Fig.1k. (b)-(e) Number of animals (SNAP25-G6s) = 5. Scale bar denotes 1 mm.
f. Average peak amplitude before/after saline injection. Similar to Figure 1d.
g. Event frequency before/after saline injection. Similar to Figure 1e.
h. Global bilateral correlation before/after saline injection. Similar to Figure 1j.
i. Seed-based bilateral correlation before/after saline injection. Similar to Figure 1k. (f)-(j) Number of animals (SNAP25-G6s) = 5. Scale bar denotes 1 mm.

n.s. $p > 0.05$, ∗ $p < 0.05$, ∗∗ $p < 0.01$, ∗∗∗ $p < 0.001$, **** $p < 0.0001$, two-tailed unpaired t test with Welch's correction. (b) $p = 0.752$ (c) $p = 0.8557$ (d) $p = 0.0005$ (e) p values of Low, Mid, High, Averaged: 0.001197, 0.00082, 0.001811, 0.000755 (g) $p = 0.1574$ (h) $p = 0.3523$ (i) $p = 0.8762$ (j) p values of Low, Mid, High, Averaged: 0.56839, 0.889722, 0.682008, 0.792959. Source data and exact p values are provided as a Source Data file.

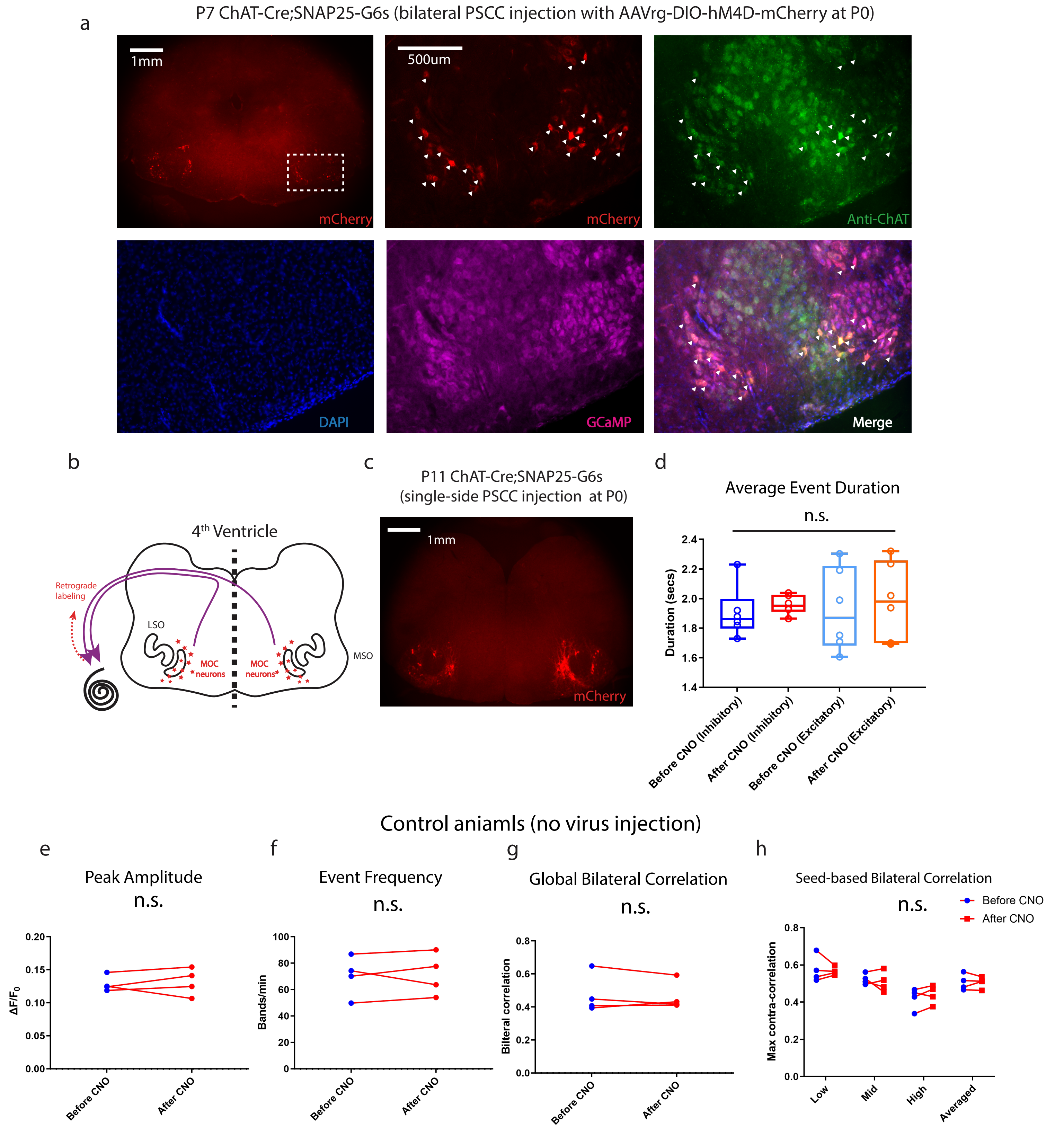

Supplementary Fig. 5: Verification of DREADD expression and chemogenetics control experiments (related to Fig. 4)

Supplementary Fig.5. Verification of DREADD expression and chemogenetics control experiments (related to Fig. 4)

a. A brainstem section that contains olivocochlear neurons expressing mCherry. Magnification: 2.5X. Scale bar: 1mm. Following panels: high magnification images of the white dashed rectangle in the first panel: showing mCherry, Alexa 647, DAPI, EGFP channels, and the merged image. Magnification: 10x. Scale bar: 500um. Experimental information: ChAT-Cre;SNAP25-G6s animal was perfused at P7 (bilateral PSCC injection with AAVrg-DIO-hM4D-mCherry at P0). White arrowheads denote cells that express mCherry/infected by the retrograde virus.
b. Schematics of cochlea receiving bilateral efferent feedback from the medial olivocochlear neurons (MOC). LSO: lateral superior olive; MSO: medial superior olive.
c. A brainstem section that contains olivocochlear neurons expressing mCherry. Magnification: 2.5X. Scale bar: 1mm. Experimental information: ChAT-Cre;SNAP25-G6s animal was perfused at P11 (single-side PSCC injection to the left cochlea with AAVrg-DIO-hM3D-mCherry at P0).
d. Average event frequency before and after chemogenetics manipulations.
e. Average peak amplitude before/after CNO in controls (no virus injection). Similar to Figure 1D.
f. Event frequency in control animals. Similar to Figure 1e.
g. Global bilateral correlation in control animals. Similar to Figure 1j.
h. Seed-based bilateral correlation in control animals. Similar to Figure 1k. (f)-(j) Number of animals (SNAP25-G6s) = 4.

Significance marks: n.s. $p > 0.05$, ∗ $p < 0.05$, ∗∗ $p < 0.01$, ∗∗∗ $p < 0.001$, **** $p < 0.0001$, two-tailed unpaired t test with Welch's correction. (e) $p = 0.8017$ (f) $p = 0.9226$ (g) $p = 0.8811$ (h) p values of Low, Mid, High, Averaged: 0.816893, 0.652684, 0.613872, 0.968344. Source data and exact p values are provided as a Source Data file. For (a), Immunohistochemistry was confirmed on 2 P7 animals (bilateral PSCC injection with AAVrg-DIO-hM4D-mCherry at P0) and 2 P11 animals (bilateral PSCC injection with AAVrg-DIO-hM3D-mCherry at P0-P1). For (c), mCherry expression was confirmed in 2 P11 animals (single-side PSCC injection with AAVrg-DIO-hM3D-mCherry at P0-P1).

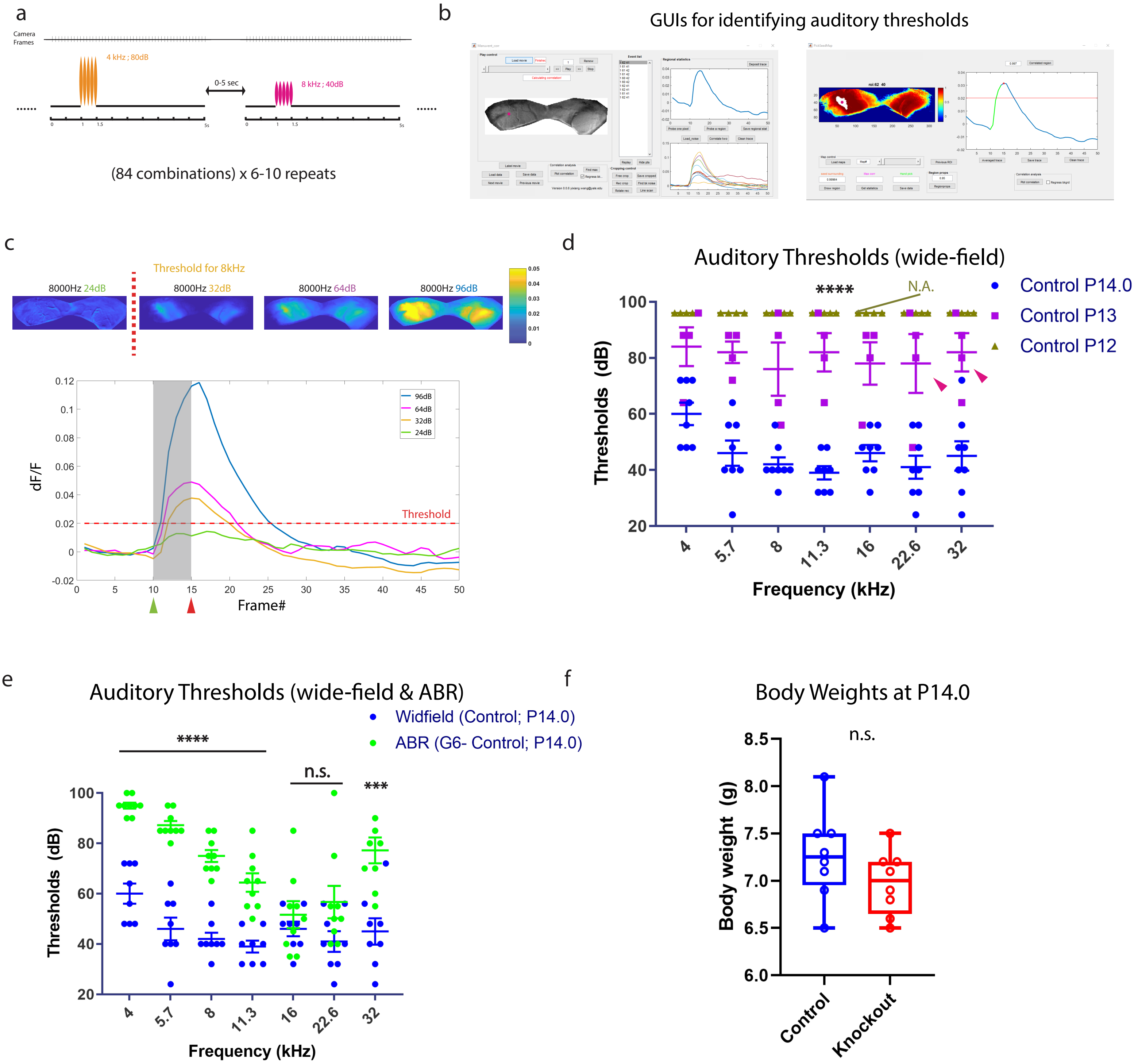

Supplementary Fig. 6: Wide-field calcium imaging resolves auditory thresholds at lower levels (related to Fig. 5 and Methods)

Supplementary Fig.6. Wide-field calcium imaging resolves auditory thresholds at lower levels (related to Fig.5 and Methods)

a. Diagram illustrating scheme of acoustic stimuli. Ticks on the "Camera Frames" axis represent endpoints of every frame. Each trial contains 5 seconds of activity/50 frames (acquired at 10 Hz). SAM tones illustrated as wave packets. Note that heights of wave packets are disproportionate to actual voltage amplitudes. See also Methods. Sec/s: second. See also Methods.
b. GUIs "Manuvent" and "PickSeedMap" are compatible to identify auditory thresholds.
c. Top panels: example $\Delta F/F_0$ responses to 8kHz tones at four different decibel levels. Colormap: parula (MATLAB). Bottom panel: corresponding $\Delta F/F_0$ curves. Dashed red line indicates threshold. Shaded area denotes the period during which acoustic stimuli are presented.
d. Auditory thresholds measured by wide-field imaging in control animals at postnatal day 12,13, and 14.0. Number of animals: Control P14.0 group = 8 (same animals as in Fig.5e, wide type SNAP25-G6s = 6; α9/α10 double heterozygous SNAP25-G6s = 2). Control P13 group = 5 (wide type SNAP25-G6s). Control P12 group = 4 (wide type SNAP25-G6s). N.A. indicates "not available" in the P12 group (no response at the maximum decibel level across the spectrum). Magenta arrowheads indicate that part of the data is not available in the P13 group (1, and 2 animals did not respond at 22.6, and 32 kHz respectively). Two-way ANOVA , Column factor (age), $F(2, 91) = 232.3$, **** $p < 0.0001$, between P13 group and P14.0 group at each frequency. Error bars indicate SEM.
e. Compare auditory thresholds measured by wide-field imaging and auditory brainstem response recording in control animals at P14.0. Number of animals: Widefield P14.0 group = 8 (same animals as in Fig.5e, wide type SNAP25-G6s = 6; α9/α10 double heterozygous SNAP25-G6s = 2). ABR P14.0 group = 9 (GCaMP6s negative littermates, same animals as in Fig.5B). Magenta arrowheads indicate that part of the data is not available in the ABR recording. n.s. $p > 0.05$, ***$p < 0.001$, **** $p < 0.0001$, two-tailed unpaired t test with Welch's correction conducted between two conditions at each frequency using available data. Error bars indicate SEM.
f. Body weights in grams. Control P14.0 group = 8 (same animals as in Fig.5e, wide type SNAP25-G6s = 6; α9/α10 double heterozygous SNAP25-G6s = 2). Knockout = 8 (mix of single knockout of either α9 or α10 subunit and double knockout). Box plot hinges: 25 percentile (top), 75 percentile (bottom). Box whiskers (bars): Max value (top), Min value (bottom). The line in the middle of the box is plotted at the median. Significance marks: n.s. $p = 0.1848$, two-tailed

unpaired t test with Welch's correction. Source data and exact *p* values are provided as a Source Data file.

Conditions	Mean value	P value (one-sample t-test compared to 0)
WT P11-P12 (averaged seed-based correlation) Fig.1k	0.1809	0.0003 ***
P6-7 a9a10 KO (low-frequency region) Fig.2g	0.1224	0.0387 *
P6-7 Acute Apamin (low-frequency region) Fig.3f	0.1752	0.0035 **
P6-7 Acute alpha-Bungarotoxin (averaged correlation) Supplementary Fig. 4e	0.2763	0.0086 **
P6-7 Inhibitory DREAD (averaged correlation) Fig.4f	0.3011	0.0008 ***

Supplementary Table 1. Residual bilateral correlations are significantly higher than zero

Significance marks: n.s. >0.05, ∗ $p < 0.05$, ∗∗ $p < 0.01$, ∗∗∗ $p < 0.001$. one-sample t test compared against constant 0. Source data are provided as a Source Data file.
