# Supplementary material for: Efferent feedback controls bilateral auditory spontaneous activity
Source: Nat Commun. 2021 Apr 27;12:2449. doi: 10.1038/s41467-021-22796-8 (PMC8079389; doi:10.1038/s41467-021-22796-8)
Supplement: Supplementary file 3 — Description of Additional Supplementary Files [file 41467_2021_22796_MOESM3_ESM.pdf]

Supplementary Movie 1 Spontaneous bands at different ages and comparison between automatically and manually labeled results (related to Fig. 1 and Methods)

Part I. Spontaneous bands at different ages: showing $\Delta F/F_0$ movies. A built-in Matlab function “mat2gray” was used to convert matrices of $\Delta F/F_0$ values to grayscale frames. $\Delta F/F_0$ value range [0, 0.3] was projected to grayscale [0, 1] (values smaller or equal to 0 were converted to 0, and values larger or equal to 0.3 were converted to 1) across all ages. Grayscale frames were then pseudo-colored with colormap “parula”. White ellipses delineate left and right inferior colliculi (IC). All $\Delta F/F_0$ Movies playback at 50 frames/second.

Part II. Automatic line-scan labeling vs. manually labeled results. Showing an example hemisphere of the IC. Red circles denote the identified peaks/ bands.

Supplementary Movie 2 Seed-based correlation maps with moving seeds at P6 and P12 (related to Fig. 1)

Two arrays of example seed-based correlation maps of a P6 and a P12 animals. Yellow squares denote the locations of seeds. The movie display correlation maps while seeds moving across the entire field of view (of the IC). Color limit: [-0.2, 1]. Color map: jet.

Supplementary Movie 3 Seed-based correlation maps with moving seeds for littermates with different genotype (related to Fig. 2)

a9/a10: α9/α10; Het: heterozygous; KO: knockout. Color limit: [-0.2, 1]. Color map: jet.

Supplementary Movie 4 Spontaneous activity and seed-based correlation maps before and after application of apamin (related to Fig. 3)

Part I. Comparison of spontaneous activity before/after apamin. Color limit: [0, 0.3]. Colormap: parula.

Part II. Comparison of correlation maps before/after apamin. Color limit: [-0.2, 1]. Color map: jet.

Supplementary Movie 5 Seed-based correlation maps before and after application of CNO (related to Fig. 4)

Part I. Inhibitory DREADD case. Color limit: [-0.2, 1]. Color map: jet.

Part II. Excitatory DREADD case. Color limit: [-0.2, 1]. Color map: jet.

Supplementary Movie 6 Tonotopic auditory responses in the dorsal IC and calcium responses to different sound levels (related to Fig. 5)

Part I. Tonotopic auditory responses in the dorsal IC. Color limit: [0, 0.3]. Colormap: parula.

Part II. IC calcium responses to different sound levels. Color limit: [0, 0.3]. Colormap: parula.

Movies playback at 50 frames/second.
