# Supplementary material for: Efferent feedback controls bilateral auditory spontaneous activity
Source: Nat Commun. 2021 Apr 27;12:2449. doi: 10.1038/s41467-021-22796-8 (PMC8079389; doi:10.1038/s41467-021-22796-8)
Supplement: Supplementary file 5 — Reporting Summary [file 41467_2021_22796_MOESM5_ESM.pdf]

nature research

Corresponding author(s): Michael Crair
Last updated by author(s): 02/11/2021

Reporting Summary

Nature Research wishes to improve the reproducibility of the work that we publish. This form provides structure for consistency and transparency in reporting. For further information on Nature Research policies, see our Editorial Policies and the Editorial Policy Checklist.

Statistics

For all statistical analyses, confirm that the following items are present in the figure legend, table legend, main text, or Methods section.

n/a	Confirmed	
☐	☒	The exact sample size (*n*) for each experimental group/condition, given as a discrete number and unit of measurement
☐	☒	A statement on whether measurements were taken from distinct samples or whether the same sample was measured repeatedly
☐	☒	The statistical test(s) used AND whether they are one- or two-sided<br>*Only common tests should be described solely by name; describe more complex techniques in the Methods section.*
☐	☒	A description of all covariates tested
☐	☒	A description of any assumptions or corrections, such as tests of normality and adjustment for multiple comparisons
☐	☒	A full description of the statistical parameters including central tendency (e.g. means) or other basic estimates (e.g. regression coefficient) AND variation (e.g. standard deviation) or associated estimates of uncertainty (e.g. confidence intervals)
☐	☒	For null hypothesis testing, the test statistic (e.g. *F*, *t*, *r*) with confidence intervals, effect sizes, degrees of freedom and *P* value noted<br>*Give P values as exact values whenever suitable.*
☒	☐	For Bayesian analysis, information on the choice of priors and Markov chain Monte Carlo settings
☒	☐	For hierarchical and complex designs, identification of the appropriate level for tests and full reporting of outcomes
☐	☒	Estimates of effect sizes (e.g. Cohen's *d*, Pearson's *r*), indicating how they were calculated

Our web collection on statistics for biologists contains articles on many of the points above.

Software and code

Policy information about availability of computer code

Data collection	Details of how the following softwares/systems were used are described in the Methods section.<br>1. ZEN Digital Imaging for Light Microscopy (Zeiss) RRID:SCR_013672<br>2. Behavioral Auditory PHYsiology (BAPHY, Open-source software from the Neural Systems Laboratory, University of Maryland, College Park) Available at: https://bitbucket.org/lbhb/baphy/src/master<br>3. BioSigRP (Tucker-Davis Technologies) RRID:SCR_014590<br>4. SPIKE2 (Cambridge Electronic Device) RRID:SCR_000903<br>5. Master 8 (multi-channel neurophysiological stimulator, A.M.P.I.) www.ampi.co.il<br>6. USG Electret Ultrasound Microphone and UltraSoundGate 116Hb (Avisoft Bioacoustics)<br><br>Software version:<br>BAPHY Version 1.00<br>BioSigRP Version 4.4.10<br>UltraSoundGate Version 4.2<br>Microphone (USM EK-FG USG)
Data analysis	1. All custom scripts/softwares (MATLAB graphic user interfaces) developed/used in the paper is available at: https://github.com/CrairLab:<br>*Object-oriented pre-processing pipeline for wide-field auditory data analysis (10.5281/zenodo.4516001, this Paper) https://github.com/CrairLab/Yixiang_OOP_pipeline<br>*Graphic-user-interface for wide-field auditory data analysis (10.5281/zenodo.4515995, this Paper) https://github.com/CrairLab/Manuvent<br>*Graphic-user-interface for seed-based correlation analysis (10.5281/zenodo.4515999, this Paper) https://github.com/CrairLab/PickSeedMap

*Graphic-user-interface for dimensionality reduction and unsupervised clustering (10.5281/zenodo.4515989, this Paper) https://github.com/CrairLab/GUI_dimReduction
Details of data analysis are described in the Methods section.

2. GraphPad Prism (RRID:SCR_002798) and Adobe Illustrator (RRID:SCR_010279) were used to generate/organize figures.

For manuscripts utilizing custom algorithms or software that are central to the research but not yet described in published literature, software must be made available to editors and reviewers. We strongly encourage code deposition in a community repository (e.g. GitHub). See the Nature Research guidelines for submitting code & software for further information.

Data

Policy information about availability of data

All manuscripts must include a data availability statement. This statement should provide the following information, where applicable:

- Accession codes, unique identifiers, or web links for publicly available datasets
- A list of figures that have associated raw data
- A description of any restrictions on data availability

Source data are provided with this paper. Raw data that support the findings of this study (Figures 1-5 and Supplementary Figures 1-6) are available from the corresponding author (michael.crair@yale.edu) upon reasonable request.

Field-specific reporting

Please select the one below that is the best fit for your research. If you are not sure, read the appropriate sections before making your selection.

☒ Life sciences ☐ Behavioural & social sciences ☐ Ecological, evolutionary & environmental sciences

For a reference copy of the document with all sections, see nature.com/documents/nr-reporting-summary-flat.pdf

Life sciences study design

All studies must disclose on these points even when the disclosure is negative.

Sample size	Sample size was determined based previously published studies (Ackman JB, Burbridge TJ, Crair MC. Retinal waves coordinate patterned activity throughout the developing visual system. Nature. 2012;490(7419):219-225; Gribizis A, Ge X, Daigle TL, et al. Visual Cortex Gains Independence from Peripheral Drive before Eye Opening. Neuron. 2019;104(4):711-723 e713; Babola TA, Li S, Gribizis A, et al. Homeostatic Control of Spontaneous Activity in the Developing Auditory System. Neuron. 2018;99(3):511-524 e515) that provide guidance to the expected variability observed during experiments and an associated power analyses. Each set of experiments includes data of 6 to more than 10 neonatal animals from at least 2 different litters.
Data exclusions	1. Due to the technical difficulties of conducting in vivo brain imaging in neonates with craniotomy, for wide-field calcium imaging data: if animals underwent brain death (extensively bright whole-brain fluorescent flushes followed by an end of detectable activity) during data acquisition, brain activity data acquired within 30 minutes before brain death happened was excluded. Individuals that provided less than 30 minutes of usable brain activity data were also excluded.<br>2. Due to the technical difficulties of conducting posterior semicircular canal (PSCC) injections in neonates, for chemogenetic experiments, expression of DREADD receptors via retrograde virus infection were checked using a Zeiss Axio Imager Z2 equipped with a CCD camera (AxioCam HRC, Carl Zeiss) for all animals. Individuals that did not show detectable co-expressing mCherry signal in brain slices were excluded.<br><br>All exclusion criteria are pre-established.
Replication	All experiments in this study were replicated with several animals. Each set of experiments includes data of 6 to more than 10 neonatal animals from at least 2 different litters, numbers of animals are shown in figure legends. All attempts were successful and all data is reported in the study.
Randomization	All animals/samples were randomly allocated into groups.
Blinding	For manually labeled data (related to Supplementary Data Fig. 1) and visually identified ABR thresholds (related to Fig. 5 and Extended Data Fig. 6): Movies or ABR responses acquired under different experimental conditions were shuffled and the tester was blinded to any prior information (age, genotype, weight, sex).<br><br>For other experiments in this study: blinding was not relevant as exactly the same analysis pipeline, criteria and unbiased statistical tests were used to experiments across different conditions (genotypes, before/after certain manipulations).

Reporting for specific materials, systems and methods

We require information from authors about some types of materials, experimental systems and methods used in many studies. Here, indicate whether each material, system or method listed is relevant to your study. If you are not sure if a list item applies to your research, read the appropriate section before selecting a response.

Materials & experimental systems

n/a	Involved in the study
☐	☒ Antibodies
☒	☐ Eukaryotic cell lines
☒	☐ Palaeontology and archaeology
☐	☒ Animals and other organisms
☒	☐ Human research participants
☒	☐ Clinical data
☒	☐ Dual use research of concern

Methods

n/a	Involved in the study
☒	☐ ChIP-seq
☒	☐ Flow cytometry
☒	☐ MRI-based neuroimaging

Antibodies

Antibodies used

1. Anti-Choline Acetyltransferase (ChAT) Antibody (Goat) Millipore Cat# AB144P RRID: AB_2079751
2. Alexa Fluor® 647 AffiniPure Donkey Anti-Goat IgG (H+L) Jackson ImmunoResearch Cat# 705-605-147 RRID: AB_2340437

Validation

1. https://www.emdmillipore.com/US/en/product/Anti-Choline-Acetyltransferase-Antibody,MM_NF-AB144P#documentation
2. https://www.jacksonimmuno.com/catalog/products/705-605-147

Animals and other organisms

Policy information about studies involving animals; ARRIVE guidelines recommended for reporting animal research

Laboratory animals

SNAP25-G6s animals: B6.Cg-Snap25tm3.1Hze/J (JAX#025111) animals that have pan-neuronal GCaMP6s expression were used widely in this study.

SNAP25-G6s animals: B6.Cg-Snap25tm3.1Hze/J (JAX#025111) animals that have pan-neuronal GCaMP6s expression were used widely in this study. α9/α10;SNAP25-G6s animals: α9/α10 nAChR double-knockout (α9-/- α10-/-) animals on C57BL/6 background were obtained from Dr. Barbara Morley 22 (Chrna9tm1Bjmy MGI:5787807; Chrna10tm1Bjmy MGI:5787808). α9-/- α10-/- line was crossed to SNAP25-G6s line (JAX #025111) to generate double-heterozygous animals that express GCaMP6s (α9+/- α10+/-; SNAP25-G6s+/null). These animals were backcrossed to a α9-/- α10-/- line to generate four different genotypes of offspring (double-heterozygous: α9+/- α10+/-, single-knockout: α9+/- α10-/-, α9-/- α10+/-, double-knockout: α9-/- α10-/-) with or without GCaMP6s expression (SNAP25-G6s+/null or SNAP25-G6snull/null). ChAT-Cre;SNAP25-G6s animals: Homozygous ChAT-Cre+/+ animals (JAX #018957) were crossed to SNAP25-G6s animal (JAX #025111) to generate ChAT-Cre heterozygous offspring that express GCaMP6s (ChAT-Cre+/-; SNAP25-G6s+/null).

ChAT-Cre;SNAP25-G6s animals: Homozygous ChAT-Cre+/+ animals (JAX #018957) were crossed to SNAP25-G6s animal (JAX #025111) to generate ChAT-Cre heterozygous offspring that express GCaMP6s (ChAT-Cre+/-; SNAP25-G6s+/null).

Animals of both sexes were used in this study. Animal care and use followed the Yale Institutional Animal Care and Use Committee (IACUC), the US Department of Health and the Human Services, and institution guidelines. All animals were housed in a regulated environment (temperature: 70 degree Fahrenheit; Humidity: 50-60%; Dark/light cycle: 12:12). In Fig. 1, SNAP25-G6s animals between P0-P13 were used for spatiotemporal and correlation analysis at different ages. In Fig. 2 and related Extended Data Fig. 3, GCaMP6s-positive α9/α10;SNAP25-G6s animals (see Transgenic Models) were used for spatiotemporal and correlation analysis at P6-P7 or at P3-4. In Fig. 3 and related Extended Data Fig. 2, SNAP25-G6s animals between P5-7 were used for in vivo pharmacological experiments. In Fig. 4, GCaMP6s-positive ChAT-Cre;SNAP25-G6s animals were used for chemogenetic experiments. In Fig. 5 and the related Extended Data Fig. 4, the control group consists of wide-type SNAP25-G6s animals and α9/α10 double heterozygous animals. The α9/α10 knockouts consists of single and double knockout of α9 and/or α10 subunits. GCaMP6s positive animals were used for auditory thresholds measurement with wide-field imaging. Their GCaMP6s negative littermates were used for auditory brainstem response.

Wild animals

No wild animals were involved in this study.

Field-collected samples

No field-collected samples were involved in this study.

Ethics oversight

Animal care and use followed the Yale Institutional Animal Care and Use Committee (IACUC), the US Department of Health and the Human Services, and institution guidelines.

Note that full information on the approval of the study protocol must also be provided in the manuscript.
